# Chronic Treatment with Novel Small Molecule Hsp90 Inhibitors Rescues Striatal Dopamine Levels but Not α-Synuclein-Induced Neuronal Cell Loss

**DOI:** 10.1371/journal.pone.0086048

**Published:** 2014-01-20

**Authors:** Nikolaus R. McFarland, Hemi Dimant, Laura Kibuuka, Darius Ebrahimi-Fakhari, Cody A. Desjardins, Karin M. Danzer, Michael Danzer, Zhanyun Fan, Michael A. Schwarzschild, Warren Hirst, Pamela J. McLean

**Affiliations:** 1 Center for Translational Research in Neurodegenerative Disease, Department of Neurology, University of Florida, Gainesville, Florida, United States of America; 2 MassGeneral Institute for Neurodegenerative Disease, Department of Neurology, Massachusetts General Hospital, Charlestown, Massachusetts, United States of America; 3 Division of Neurology and Inherited Metabolic Diseases, Children’s Hospital, Heidelberg University Hospital, Ruprecht-Karls University Heidelberg, Heidelberg, Germany; 4 Deparment of Neurology, Universitatsklinikum Ulm, Ulm, Germany; 5 Pfizer Neuroscience Research Unit, Cambridge, Massachusetts, United States of America; 6 Department of Neuroscience, Mayo Clinic, Jacksonville, Florida, United States of America; Hokkaido University, Japan

## Abstract

Hsp90 inhibitors such as geldanamycin potently induce Hsp70 and reduce cytotoxicity due to α-synuclein expression, although their use has been limited due to toxicity, brain permeability, and drug design. We recently described the effects of a novel class of potent, small molecule Hsp90 inhibitors in cells overexpressing α-synuclein. Screening yielded several candidate compounds that significantly reduced α-synuclein oligomer formation and cytotoxicity associated with Hsp70 induction. In this study we examined whether chronic treatment with candidate Hsp90 inhibitors could protect against α-synuclein toxicity in a rat model of parkinsonism. Rats were injected unilaterally in the substantia nigra with AAV8 expressing human α-synuclein and then treated with drug for approximately 8 weeks by oral gavage. Chronic treatment with SNX-0723 or the more potent, SNX-9114 failed to reduce dopaminergic toxicity in the substantia nigra compared to vehicle. However, SNX-9114 significantly increased striatal dopamine content suggesting a positive neuromodulatory effect on striatal terminals. Treatment was generally well tolerated, but higher dose SNX-0723 (6–10 mg/kg) resulted in systemic toxicity, weight loss, and early death. Although still limited by potential toxicity, Hsp90 inhibitors tested herein demonstrate oral efficacy and possible beneficial effects on dopamine production in a vertebrate model of parkinsonism that warrant further study.

## Introduction

Protein aggregates such as beta amyloid in Alzheimer’s disease, tau deposits in frontotemporal dementia, and Lewy bodies in Parkinson disease (PD) are a common pathological feature in neurodegenerative disorders. Molecular chaperones, such as heat shock proteins, co-localize with aggregates in neurodegenerative disease and play a critical role in protein processing and homeostasis [1], [2]. Heat shock proteins (Hsp) such as Hsp70 direct misfolded and potentially toxic proteins for degradation via the proteasome or autophagy-lysosomal system [3]–[5]. Furthermore, induction of Hsp70 is protective in models of neurodegenerative disorders, such as Huntington’s disease, spinocerebellar ataxias, and tauopathy disorders (i.e., Alzheimer’s disease) [6]–[8]. We and others have demonstrated that Hsp70 can enhance the degradation of misfolded α-synuclein, reduce oligomer formation, and mediate toxicity due to α-synuclein overexpression [9]–[11]. Moreover, direct pharmacological upregulation of Hsp70 with geldanamycin, an Hsp90 inhibitor, results in decreased cytotoxicity from α-synuclein [12]. Thus targeting molecular chaperones, such as Hsp70 or Hsp90, has reasonable therapeutic potential not only for parkinsonism, but also for related neurodegenerative disorders.

A number of small molecule inhibitors of Hsp90 have been tested in models of PD and other neurodegenerative disorders [13], [14]. Hsp90 negatively regulates Hsp70 expression by blocking activation of the transcription factor HSF-1; thus inhibitors result in Hsp70 induction [15]. Geldanamycin is a naturally occurring benzoquinone that blocks Hsp90 interaction with HSF-1 resulting in enhanced Hsp70 expression [16]. However, its utility is limited by hepatotoxicity and poor brain permeability. In contrast, the analogues 17-(allylamino)-17-demethoxygeldanamycin (17-AAG) and 17-dimethylaminoethylamino-17-demethoxy-geldanamycin (17-DMAG) have greater potency, reduced toxicity, and cross the blood brain barrier more efficiently [6], [17]. Preliminary testing also showed neuroprotection in models of polyglutamine disorders. However, despite promising effects in clinical trials for cancer, these compounds have been pursued only in a limited fashion due to hepatotoxicity, poor oral bioavailability, and formulation issues [18], [19].

Recently, a novel class of Hsp90 inhibitors with structure different from that of geldanamycin and derivatives was discovered among a screen for drugs that bind the ATP pocket of Hsp90. SNX-2112 (4-[6,6-dimethyl-4-oxo-3-(trifluoromethyl)-4,5,6,7-tetrahydro-1H-indazol-1-yl]-2-[(trans-4-hydroxycyclohexyl)amino]benzamide; PF-04928473) was the initial drug described and exhibited potent Hsp90 inhibition, anti-tumor activity, blood-brain permeability, and oral bioavailability [20], [21]. We recently tested compounds from the same class in a PD cell model [22]. Several of these novel Hsp90 inhibitors, in particular SNX-0723 (PF-04924868), significantly reduced α-synuclein oligomer formation and cytotoxicity concomitant with Hsp70 induction. SNX-0723 also exhibited favorable pharmacokinetic properties and induced Hsp70 in rat brain [22]. Based on these findings we next wanted to test the effect of these novel Hsp90 inhibitors in a rat model of parkinsonism. We and others have demonstrated that AAV expression–utilizing a variety of viral serotypes: 1, 2, 5, 6, and 8–of α-synuclein results in progressive, dopaminergic nigrostriatal neurodegeneration over the course of several weeks [23]–[25]. This model allowed us to test whether chronic oral administration of novel Hsp90 inhibitors in rats could protect against progressive α-synuclein-induced nigrostriatal toxicity.

## Methods

### Viral Production

Construction of rAAV vectors used to express human wild-type α-synuclein was as previously described (AAV-CBA-Syn-WPRE construct) [26]. Recombinant AAV2/8 virus was generated by the Harvard Gene core (Harvard Gene Therapy Initiative, Harvard Medical School) via tripartite transfection of the *cis*-transgene, packaging (*rep* and *cap*) genes, and helper plasmid into HEK 293A cells. Viral particles were purified by iodixanol density gradient, isolated, and titered by dot blot hybridization. Final titer for rAAV expressing human α-synuclein was 5.6×10^12^ gc/mL.

### Stereotaxic Surgery and Drug Treatment

Animal protocols and procedures were approved by the MGH Subcommittee on Research Animal Care (IACUC #2005N000156) and followed recommendations in the Guide for the Care and Use of Laboratory Animals of the National Institute of Health. All surgery was performed under ketamine/xylazine anesthesia, and all efforts were made to minimize suffering. Male Sprague Dawley rats (300–350 g) were anesthetized, skull exposed, and then unilaterally injected in the substantia nigra (SN) with rAAV2/8 expressing human α-synuclein as previously described [23]. Each rat was injected with 2 µL of rAAV2/8 (1.12×10^10^ viral genomes) at 0.4 µL/min using a microinjection pump (Stoelting Co., Wood Dale, IL) with 10 µL Hamilton syringe and 33-gauge needle. After injection the syringe remained in situ for 5 minutes before withdrawal. The scalp was sutured and animals were monitored until fully recovered.

**Figure 1:**
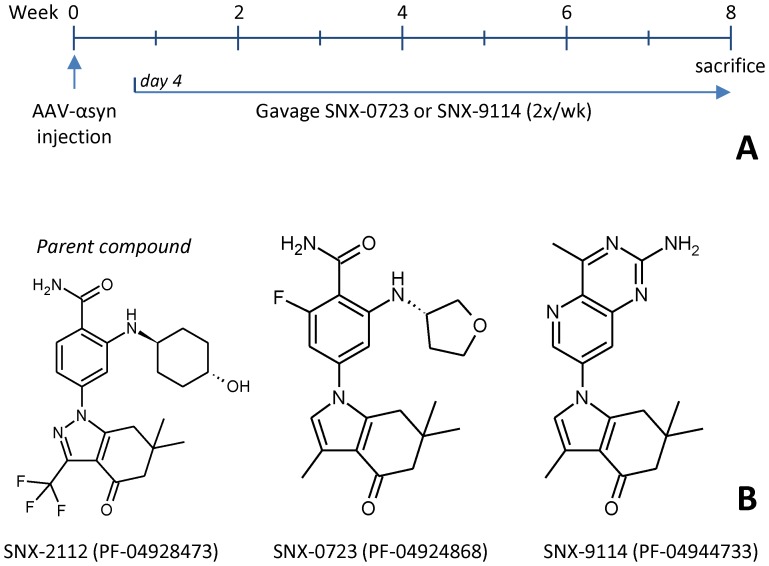
Study paradigm and structure of compounds. **A**) Illustrates the study paradigm and timecourse for drug testing in animals. **B**) Shows the structures of parent and the two derivative SNX compounds used in experiments.

Four days following recovery from surgery, rats began receiving drug or vehicle (0.5% methylcellulose) by oral gavage on a biweekly basis. Figure 1 illustrates the experimental paradigm and structures for each compound. Drug groups included SNX-0723 (PF-04924868) at 10 mg/kg and SNX-9114 (PF-04944733) at 1.5 mg/kg and 3 mg/kg. All rats were weighed routinely prior to surgery, and then at each treatment session for the duration of the experiment. Rats treated with 10 mg/kg SNX-0723 showed toxicity characterized by weight loss and failure to thrive, thus mid-treatment the dose was reduced to 6 mg/kg dose (see results).

### Tissue Preparation and Immunohistochemistry

Eight weeks post-injection, rats were deeply anesthetized and transcardially perfused with cold 0.01M phosphate buffered saline (PBS, pH 7.4) followed by 4% paraformaldehyde in PBS. Brains from a subset of animals were harvested fresh, without fixation, and the cortex, striatum, and midbrain quickly dissected on ice, snap-frozen in isopentane, and kept at −80°C for use in biochemical analyses. Perfused brains were postfixed 24 hours, then cryoprotected in 30% sucrose/PBS, and serially sectioned at 40 µm with a sliding microtome. For immunohistochemistry, free-floating sections were rinsed with PBS, then treated with endogenous peroxidase inhibitor (10% methanol and 3% H_2_O_2_), permeablized with 0.3% Triton X-100 in PBS, and blocked in 5% normal goat serum. Coronal sections through the striatum and SN were immunostained with primary antibodies to TH (1:10,000 dilution; Millipore, Billerica, MA) or α-synuclein LB509 (1:1000 dilution; Zymed Laboratories, Inc., San Francisco, CA) overnight at 4°C. After rinsing, immunostaining was visualized with biotinylated secondary, followed by avidin-biotin (Vectastain Elite Kit), and 3,3′-diaminobenzidine reaction. Immunostained sections were washed and mounted on Superfrost slides, and then counterstained with 0.05% cresyl violet per standard protocols and coverslipped (Permount, Sigma Chemicals).

### Microscopy and Stereology

Immunostained sections were viewed using an Olympus BX51 microscope, and photomicrographs were taken with Olympus DP70 digital camera and adjusted for suitable contrast and brightness. Cases in which the AAV injection was improperly placed in the SN (missed target) and poor expression of α-synuclein in the nigrostriatal system were excluded from analyses. Nigrostriatal cell loss was assessed using unbiased stereology according to the optical fractionator principle [27] as previously described [23]. The examiner was blinded to treatment group. Cell counts included the injected side compared to the uninjected contralateral SN as control. At least 8 sections (240 µm apart) though the SN for each case were analyzed and counted using the Olympus CAST Stereology System. Sampling frequency was sufficient for a coefficient of error of less than 0.1.

### Immunoblotting

Striatal and midbrain tissues were separately suspended in 8×volume/wet weight tissue of lysis buffer (50 mM Tris-HCL, pH 7.4; 175 mM NaCl; 5 mM EDTA, pH 8.0; and protease inhibitor, Roche Inc.) and homogenized on ice for 10–15 seconds with Teflon pestle. A 100 µL aliquot of this tissue suspension was removed for HPLC and treated with 100 mM H_3_PO_4_ plus 100 µM methyldopa (internal standard for HPLC recovery). Each sample was centrifuged for 15 minutes at 4°C, filtered (0.22 µm Spin-X filter, Corning, NY), and then 1% Triton X-100 added to the non-HPLC lysate. Lysates were then centrifuged for 60 min at 4°C to collect the triton-X insoluble fraction. Triton-soluble lysate was separated and the insoluble pellet resuspended in 2% SDS-containing lysis buffer (Triton-insoluble fraction), then sonicated for 10 s. Protein concentration for each lysate was determined by BCA assay. Samples were separated on a 4–12% Bis-Tris NuPage pre-cast gel (Invitrogen) with MES buffer, transferred to PVDF, and immunoblotted for Hsp70 (rabbit anti-Hsp70, Stressgen), tyrosine hydroxylase (TH; mouse TH-2 antibody, Sigma), or α-synuclein (mouse Syn1, BD Transduction Laboratories, or Syn (LB509) antibody, Zymed). All blots were immunostained for GAPDH or actin as loading control. Immunoblotted α-synuclein, TH and GAPDH were detected with secondary antibody conjugated to HRP and reacted with ECL (GE Healthcare), per protocol. Films were digitally scanned and analyzed with ImageJ software (NIH). TH and α-synuclein content for each sample was normalized to loading control.

### Hsp70 ELISA

Quantitative analysis of Hsp70 levels in rat cortical (or striatal) tissues after treatment with Hsp90 inhibitors was performed using ELISA (Stressgen, Ann Arbor, MI, USA) according to the manufacturer’s instructions and similar to that detailed by Danzer et al. [10]. Hsp70 concentrations in tissues were determined by generating a standard curve with calibrated Hsp70 protein standard and then interpolating absorbance readings using fitting software (Graph Pad 5.0).

### Dopamine Content

Striatal tissues were thawed, weighed, homogenized, and mixed in lysis buffer with methyldopa added as an internal control as described above. Dopamine (DA) and 3,4-dihydroxyphenlyacetic acid (DOPAC) were measured by HPLC with electrochemical detection and normalized to protein content per sample [28].

### Statistics

All data are expressed as group mean ± SEM. Stereological estimates of nigral TH cell survival were analyzed using one-way ANOVA with Tukey’s multiple comparison post-hoc (Prism GraphPad 5.0, San Diego, CA). Dopamine and DOPAC content were analyzed with repeated measures ANOVA and Bonferroni multiple comparisons posthoc and Spearman’s correlation. Alpha was 0.05 for all tests.

## Results

**Figure 2:**
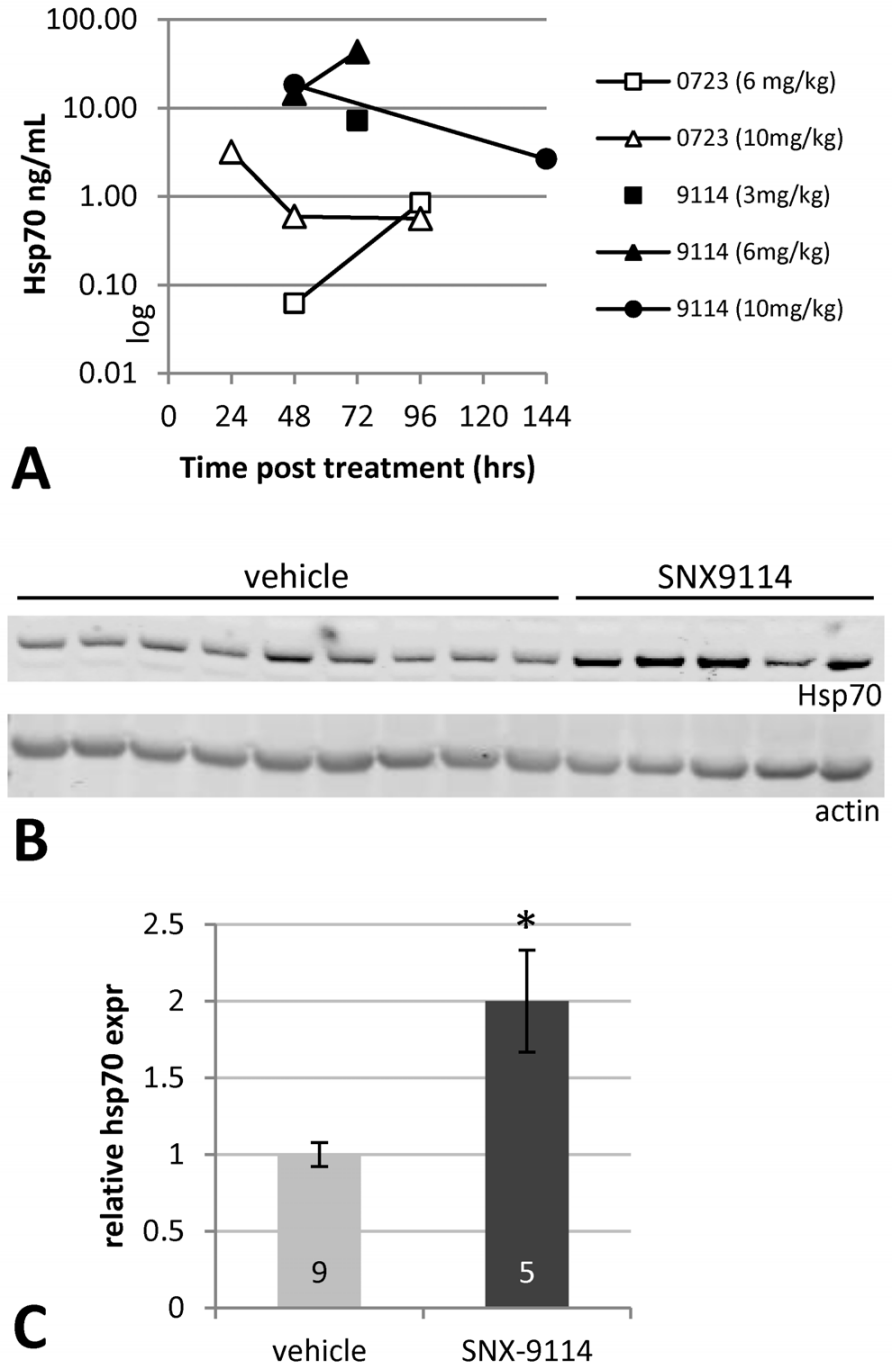
Hsp70 induction in brain. **A**) Graph of hsp70 ELISA data from cortical tissue lysates after treatment of rats with novel small molecule Hsp90 inhibitors. Tissue was harvested at 1–6 days post treatment and shows sustained hsp70 induction at ≥72 hrs for both SNX-0723 and SNX-9114. **B**) Western blot of striatal tissue homogenates from rats injected with WT α-synuclein and treated with SNX9114 (n = 5) or vehicle (n = 9), immunoblotted for hsp70 and actin as loading control. **C**) Densitometry shows significant (p = 0.037) striatal Hsp70 induction following treatment with SNX-9114.

Preliminary testing of SNX-0723 in rats at doses 10 mg/kg or higher showed lasting induction of Hsp70 in brain at least 24 hours post oral gavage (Figure 2A). The newer compound SNX-9114 likewise demonstrated excellent brain permeability and even greater potency than SNX-0723 in terms of Hsp70 induction. Limited dose-response testing also suggested more prolonged Hsp70 induction, 72 hours or greater, in brain for both Hsp90 inhibitors. Based on these findings we compared the effects of chronic oral treatment of rats with SNX-0723 at 10 mg/kg versus SNX-9114 at 1.5 and 3 mg/kg for 7–8 weeks post injection of AAV-α-synuclein. Western blot analysis of striatal extracts collected 3–4 days post final treatment confirmed a sustained 2-fold increase in Hsp70 induction in SNX-9114 treatment groups compared to vehicle (Figure 2B, C).

### Tolerability and Toxicity

**Figure 3:**
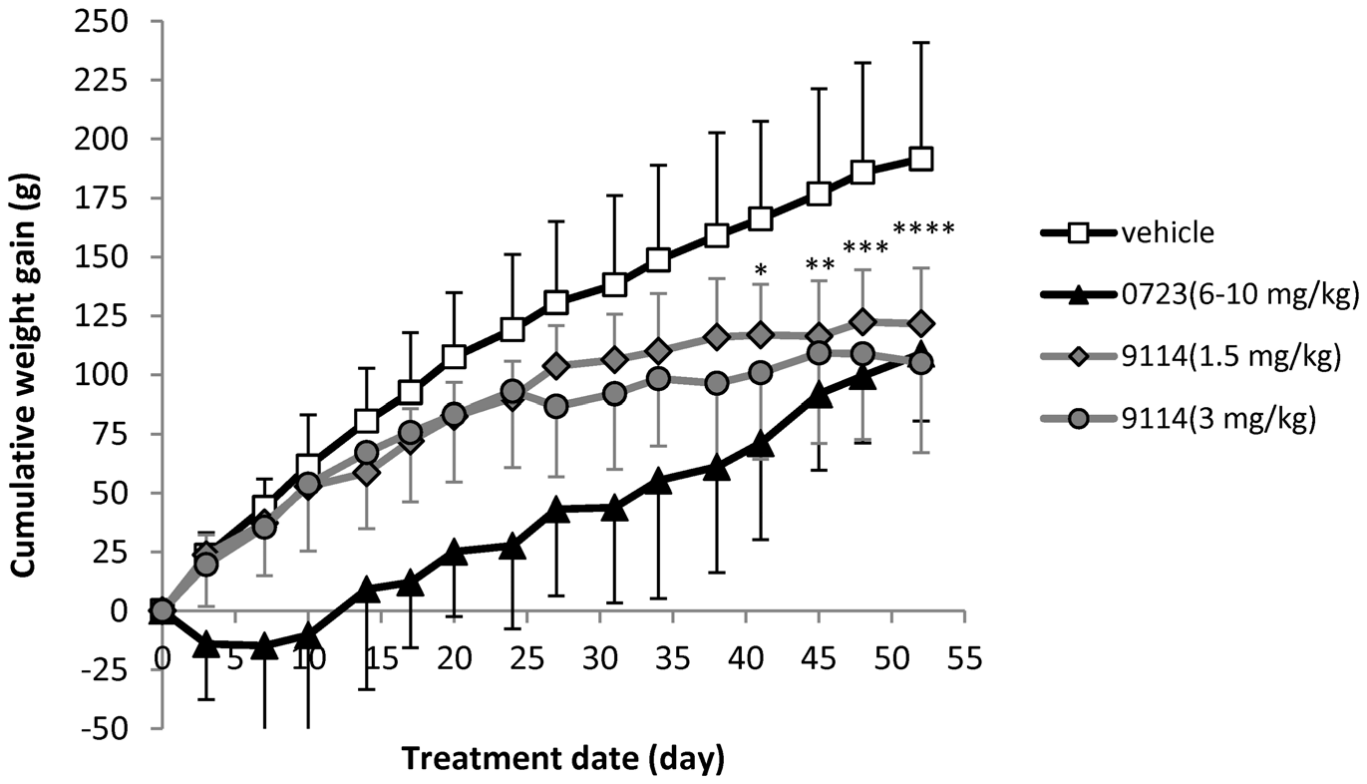
Graph of cumulative weight gain for treatment groups. SNX-0723 caused the most toxicity, weight loss, and failure to thrive at the higher dose of 10 mg/kg. All treatment groups gained weight at lower doses, including SNX-9114, but not at the rate of vehicle control (*p<0.05, **p<0.01, ***p<0.001, ****p<0.0001 for comparison of vehicle and 9114 at 1.5 mg/kg; 2-way ANOVA with Bonferroni correction). (n  = 14, 14, 12, 10, and 14 for vehicle, 0723 [3mg/kg], 0723 [6–10 mg/kg], 9114 [1.5 mg/kg] and 9114 [3 mg/kg], respectively).

Although chronic treatment with SNX-9114 was generally well tolerated, SNX-0723 at 10 mg/kg resulted in toxicity manifest by diarrhea, weight loss, failure to thrive, and early death in 7 of 21 animals. As a result, the dose of SNX-0723 was reduced mid-treatment to 6 mg/kg for all remaining rats in this group. Dose reduction was effective in reducing toxicity, reversing weight loss and mortality. However, rats did not gain weight at the same rate as vehicle control animals (Figure 3). Similarly, although rats treated with 3 mg/kg SNX-9114 did not show overt signs of toxicity, weight gains were less than that of control. Halving the SNX-9114 dose to 1.5 mg/kg in a separate treatment group made little difference in weight gain. No overt behavioral changes were observed in either of the groups or treatment regimens.

### Effects of Hsp90 Inhibitors on Nigrostriatal Toxicity

**Figure 4:**
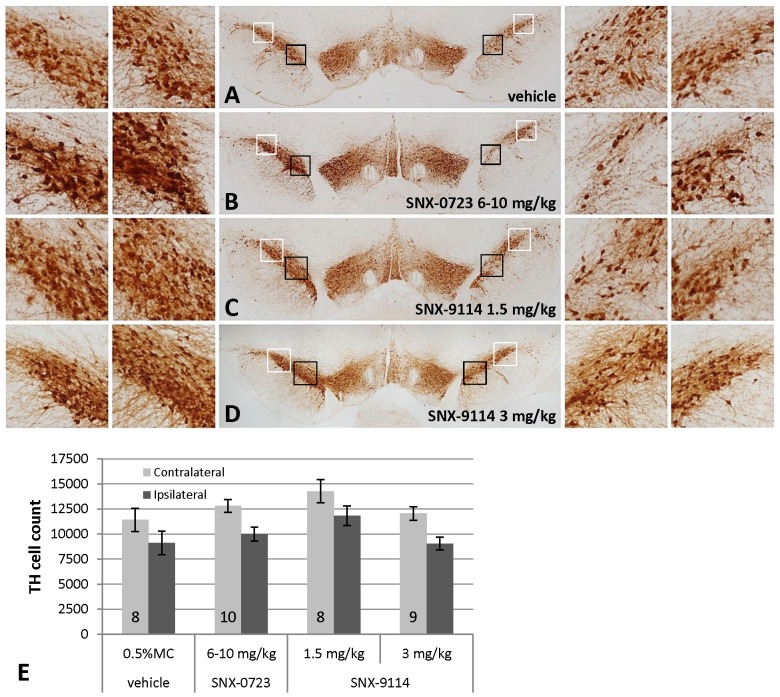
Comparison of higher dose SNX-0723 and SNX-9114 effects on nigrostriatal toxicity. A–D ) Photos show low power images of injected SN (right) and contralateral uninjected side (left) for each drug treatment group. Black (medial) and white (lateral) squares indicate regions of interest for higher magnification photos shown. There is modest cell loss on the side of the lesion for all treatments. **E**) Graph of stereological counts (mean ±SEM) of TH-positive cells in the SN ipsilateral and contralateral to AAV-α-synuclein lesion. Numbers at base of bars indicate N for each group. Analysis of variance revealed no significant differences among treatment groups.

**Figure 5:**
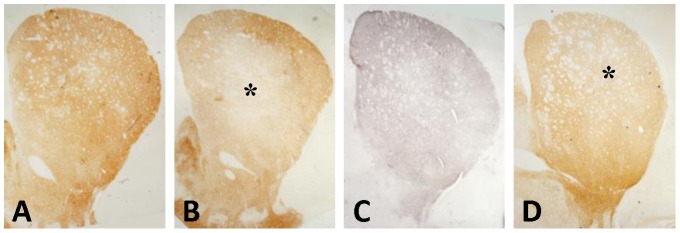
Illustration of drug effects on nigrostriatal terminal density for SNX-9114, 3 mg/kg dose. The representative photos show the distribution of TH+ terminals in the striatum contralateral and ipsilateral to AAV-α-synuclein injection in panels **A** and **B,** respectively. **C)** α-Synuclein-positive nigrostriatal terminals ipsilateral to AAV injection in same case. **D**) Photo of TH+ terminals in striatum from animal treated with lower dose SNX-9114, 1.5 mg/kg. *Marks region of TH terminal loss due to α-synuclein toxicity.

Chronic treatment with SNX-0723 or with the more potent SNX-9114 did not rescue nigral dopaminergic neurons from α-synuclein dependent toxicity. Figure 4A-C shows the distribution of TH-immunoreactive cell loss at comparable levels of the SN for both drug and vehicle treatment after viral injection. Stereological counts revealed mean TH cell loss (relative to the contralateral unlesioned SN) of 21.1%±3.7 for vehicle, 21.6%±5.0 for 6–10 mg/kg SNX-0723, and 17.0%±3.2 and 24.1%±4.7for 1.5 and 3 mg/kg doses of SNX-9114, respectively (Figure 4E). Although the lower dose of SNX-9114 appeared to have slightly less TH cell loss, one-way analysis of variance revealed no significant differences among treatment groups (*F* [3], [31] = 0.42, p = 0.39). Similarly, analysis of striatal TH terminal density as shown in representative cases (Figure 5) demonstrated no differences between drug and vehicle control groups. Likewise, among different treatment groups there was also no apparent change in α-synuclein-positive inclusion-like structures in nigrostriatal terminals or cell bodies.

### Hsp90 Inhibitor Effects on Dopamine Terminals

**Figure 6:**
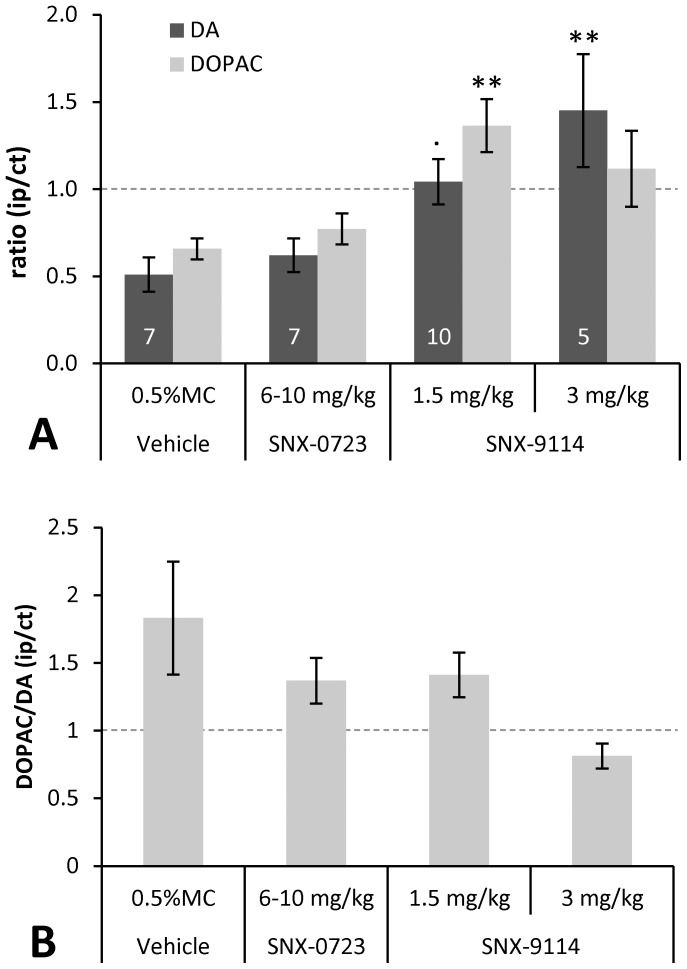
Striatal DA and DOPAC content and turnover. **A**) Graph shows the mean (±SEM) DA or DOPAC content in the striatum ipsilateral (ip) to AAV-α-synuclein injection normalized to the contralateral (ct) uninjected side (ratio ip/ct). Repeated measures ANOVA (DA and DOPAC) showed a main effect for drug (F[3], [25] = 7.05, p = 0.001) and interaction with DA metabolites (*F* [3], [25] = 3.31, p = 0.036). DA levels were significantly increased for SNX-9114 at both 1.5 (p = 0.061) and 3 (p = 0.003) mg/kg doses compared to vehicle, whereas no change for SNX-0723. DOPAC levels increased significantly only for SNX-9114 at 1.5 mg/kg (p = 0.005), but appeared also to trend toward normal for the higher dose. N for each group is noted at base of each bar (7, 7, 10, 5, respectively). **B**) Graph of DA turnover (DOPAC/DA ratio, normalized to contralateral control) for each case shows an inverse correlation between DA level and rate of turnover. ·p = <0.1, **p<0.01.

We performed biochemical analysis of striatal DA and its metabolite, DOPAC, to gauge drug effects on nigrostriatal terminal plasticity. Striatal DA and DOPAC measurements ipsilateral (ip) to rAAV α-synuclein injection were normalized to the contralateral (ct) uninjected/unlesioned side within each animal and shown as ratio of ip/ct (Figure 6A). Repeated measures ANOVA demonstrated a significant main effect for drug (*F* [3], [25] = 7.05, p = 0.001) and interaction with DA and DOPAC measures (*F* [3], [25] = 3.31, p = 0.036). Vehicle treated animals, as expected, showed (∼50%) reduction in striatal DA ipsilateral to rAAV α-synuclein injection with mean DA ratio of 0.51±0.10 relative to that in the contralateral striatum. SNX-0723 at the 6–10 mg/kg dose did not significantly alter striatal DA levels (0.62±0.10) and was similar to vehicle control. However, treatment with SNX-9114 resulted in significant increase and trend toward normalization of striatal DA and DOPAC levels compared to vehicle. DA content for the 1.5 mg/kg dose was 1.04±0.13 (p = 0.061) and the 3 mg/kg dose 1.45±0.32 (p = 0.003). DOPAC was also significantly increased for SNX-9114 1.5 mg/kg dose, 1.36±0.15 (p = 0.005), and likewise showed a non-significant trend for normalization at 3 mg/kg dose, 1.12±0.22 (p = 0.28). We also examined an index of DA turnover to DOPAC (DOPAC/DA ratio), which negatively correlated with DA changes, *r_s_* = −0.67, p<0.01 (Figure 6B). Decreases in DA for control and SNX-0723 at 6–10 mg/kg corresponded to increase in DOPAC/DA ratio (1.57±0.2 and 1.34±0.16, respectively), or turnover. By contrast, in the case of the 3 mg/kg dose of SNX-9114 relative increase in striatal DA resulted in non-significant decrease in DOPAC/DA ratio (0.81±0.09).

## Discussion

Modulation of molecular chaperones with small molecule Hsp90 inhibitors has gained recent attention as potential novel therapeutics for parkinsonism and other neurodegenerative disorders that manifest proteinopathy [13], [29], [30]. We recently reported that novel small molecule Hsp90 inhibitors in a neuroglioma cell model of parkinsonism can reduce formation of toxic dimer/oligomeric species of α-synuclein and prevent cytotoxicity [22]. In the current study, we examined whether chronic treatment with candidate, small molecule Hsp90 inhibitors could protect against α-synuclein-induced nigrostriatal toxicity in a targeted viral model of parkinsonism in the rat. Chronic treatment twice weekly was best tolerated with SNX-9114, but neither SNX-0723 nor SNX-9114 protected against loss of dopaminergic nigrostriatal neurons in our model. Several possibilities may explain these results including length of treatment, onset of therapy, and inter-animal variability. Longer incubation with AAV-synuclein (12 vs 8 weeks, personal observation) can result in greater dopamine cell loss and, combined with chronic Hsp90 inhibitor therapy, might have increased our ability to detect potential differences between vehicle and drug groups. Although we started treatment early–4 days post viral injection when viral transgene expression is only beginning–pretreatment before AAV injection may be required in our model to prevent dopamine cell loss in the rat SN as seen in prior cell studies [22]. Variability in this model and treatment paradigm likewise could have contributed to lack of findings, indicating need for greater animal numbers to the increase the power of our observations.

Although Hsp70 expression or induction (i.e., via Hsp 90 inhibition) has been shown to reduce α-synuclein dimer/oligomers and cytotoxicity in cell models [10], [12], [31], [32], few studies have examined the effects of increased Hsp70 on α-synuclein toxicity in animal models. In *Drosophila* Hsp70 expression has been shown to reduce dopaminergic neuronal loss associated with α-synuclein [11]. Crossing Hsp70 expressing mice with transgenic mice that express human wild-type α-synuclein (line D), we subsequently demonstrated that Hsp70 specifically reduces “toxic” high-molecular weight α-synuclein species [9]. In contrast, Shimsheck et al. (2010) examined transgenic mice co-expressing both human A53T mutant α-synuclein and Hsp70(HspA1A) under the control of the Thy1 promoter and found that mice overexpressing Hsp70 actually performed worse on behavioral tests than single transgenic α-synuclein(A53T) mice [33]. Moreover, Hsp70 overexpression did not cause change in α-synuclein expression, oligomers, phosphorylation, or localization in brain. These findings are difficult to explain, but possibilities include inadequate level of Hsp70 expression, non-functional Hsp70, or lack of other co-chaperones such as Hsp40 or Hsp90 which enhance Hsp70 ATPase activity [34]. Differences in interaction between Hsp70 and wild-type vs A53T α-synuclein may also contribute but remain unclear. Besides Hsp70 other heat shock proteins may be (more) effective, such as Hsp104 which when tested in a rat model of α-synuclein overexpression reduced dopaminergic cell loss and phosphorylated α-synuclein-containing inclusions [35]. In vitro Hsp27 expression has also been shown to have more potent effect than Hsp70 on toxicity associated with mutant and wild-type α-synuclein [36]. Recent studies by Daturpalli et al. (2013) suggest also that Hsp90 itself interacts with oligomeric α-synuclein and can inhibit fibril formation and α-synuclein toxicity in SHSY5Y cells [37]. Together these data indicate need for further study of heat shock protein effects on α-synuclein in both cell and animal models.

Despite the lack of apparent rescue of nigrostriatal dopamine cells, we observed a significant drug effect on striatal DA content and metabolism. In animals treated with SNX-9114 striatal DA and DOPAC levels increased and “normalized,” suggesting a possible effect on the remaining nigrostriatal terminals and neurochemical plasticity. These preliminary findings are potentially significant as restoration of dopamine content in the striatum improves behavioral deficits in PD models and overall function in PD patients [38], [39]. Potential mechanisms for the observed increase in striatal DA include increased TH activity or L-DOPA (L-dihydroxyphenylalanine) supply, decrease in monoamine oxidase B activity, or increased terminal DA reuptake. Compensatory mechanisms for nigrostriatal injury are well-established and residual striatal terminals can compensate for nearly 80% loss of DA innervation [38], [40]. Recent data, however, suggests that α-synuclein expression negatively regulates TH activity and can affect dopaminergic neurotransmission [41]. Results in vehicle control animals are consistent with these findings and show reduced striatal DA without evidence of compensation despite relative small nigrostriatal lesion (∼21% TH cell loss). However, the cause of increase or normalization of DA levels with SNX-9114 is less clear. Although we did not measure TH activity, striatal levels of TH for treated and control animals appeared similarly reduced due to AAV-α-synuclein lesion and there was no evidence of TH terminal sprouting as seen previously in other partial lesion models [40], [42]. While Hsp70 induction has been shown to protect dopaminergic neurons against toxic insult, including α-synuclein, very little is known about the potential effects on DA production (i.e. TH activity) or metabolism [43], [44]. Further studies are needed to evaluate possible neuromodulatory effects of small molecular Hsp90 inhibitors and Hsp70 induction on dopaminergic neurons.

A major limitation of Hsp90 inhibitor therapy unfortunately has been toxicity, which was also found for the drugs used in this study [13]. Modifications to geldanamycin leading to development of the analogues 17-AGG and 17-DMAG were initially purported to reduce toxicity, mainly hepatic, and increase potency as well as brain penetration [6], [17]. Clinical trials of these compounds primarily for cancer therapy have shown some promise, but significant concerns about hepatotoxicity and delivery issues remain, limiting their use in particular for non-oncology indications [18], [19]. Recent efforts have focused on developing novel small molecule Hsp90 inhibitors, such as those studied herein which potently inhibit Hsp90, cross the blood-brain barrier, and are orally bioavailable [20], [21]. Our initial studies in rodents demonstrate that candidate drugs, administered orally, were brain permeable at concentrations used and produced lasting induction of Hsp70 in brain tissue. However, SNX-0723 given chronically at 10 mg/kg caused animals to loose significant weight, fail to thrive, and die, forcing decrease in the dose to 6 mg/kg which was better tolerated. Although the more potent SNX-9114 did not cause overt toxicity at either dose used, rats still did not gain weight at rates equivalent to vehicle treated animals. While SNX-9114 induced Hsp70 in brains, it too had an insignificant neuroprotective effect on synucleinopathy. It is tempting to speculate whether Hsp70 induction in brain had a causal relationship to weight loss/failure-to-thrive in animals, but based on prior studies it is more likely that our candidate drugs caused peripheral toxicity (i.e., hepatic, gastrointestinal) [29], [45]. No studies to our knowledge so far have linked Hsp70 (or Hsp90) to weight homeostasis or metabolism. Further studies are needed to elucidate the source of toxicity for future trials.

To date clinical trials for Hsp90 inhibitors have primarily been limited to cancer therapy, based on their selectivity for tumor cells, modulation of Hsp90 function, and binding of client proteins [29], [46]. Kamal *et al.* have suggested that the geldanamycin derivative, 17-AAG, preferentially binds Hsp90 when it is part of a multi-chaperone complex, including co-chaperones and client protein [47], [48]. Although it is unclear if novel small molecule Hsp90 inhibitors such as those used here function similarly, such selectivity may provide similar advantage for use in neurodegenerative disorders particularly due to the probable need for long-term, chronic therapy, relative to that in cancer. Our findings, however, indicate that Hsp70 induction in brain was widespread rather than limited to tissues affected by viral α-synuclein expression. Though potentially concerning, such effects in brain may actually be advantageous. Heat shock protein induction (i.e., stress response) by Hsp90 inhibition has been shown to have purported neuroprotective effects in a variety of neurodegenerative models including Huntington’s disease [7], spinocerebellar ataxias [6], tauopathies [8], and parkinsonism [10] in which pathology spreads. Neuroprotective effects of Hsp70 induction in particular include reduction in aggregate (“toxic” oligomer) formation, cellular toxicity, and apoptosis [9], [10], [49]. Thus, targeting Hsp90 and augmenting the cellular response to stressors may still be a reasonable therapeutic approach for neurodegenerative diseases.

This study represents a first attempt to examine the ability of novel small molecule Hsp90 inhibitors to protect against α-synuclein dependent nigrostriatal toxicity in mammalian model of PD. Compared to vehicle neither compound tested protected against nigral TH-cell loss; however, our results suggest possible nigrostriatal terminal effects with normalization of DA content and turnover in striatum. These results are significant as restoration of DA in the brain is an aim of current therapeutics in Parkinson disease. Although the mechanism of nigrostriatal dopamine restoration remains unclear, these findings suggest that Hsp90 inhibition may represent a potential novel therapeutic approach to Parkinson disease and related disorders. Further study of these novel small molecule Hsp90 inhibitors is warranted and must also address toxicity concerns for future trials in neurodegenerative disease.
